# Increased PrEP uptake and PrEP-RN coincide with decreased HIV diagnoses in men who have sex with men in Ottawa, Canada

**DOI:** 10.14745/ccdr.v49i06a04

**Published:** 2023-06-01

**Authors:** Abigail Kroch, Patrick O’Byrne, Lauren Orser, Paul MacPherson, Kristen O’Brien, Lucia Light, Ryu Kang, Agatha Nyambi

**Affiliations:** 1 Ontario HIV Treatment Network; 2 University of Ottawa, School of Nursing, Ottawa, ON; 3 Ottawa Public Health, Ottawa, ON; 4 University of Ottawa, Faculty of Medicine, Ottawa, ON

**Keywords:** HIV, PrEP, PrEP-RN, epidemiology, pre-exposure prophylaxis

## Abstract

**Background:**

We sought to evaluate if increased uptake of HIV pre-exposure prophylaxis (PrEP) correlated to population-level changes in human immunodeficiency virus (HIV) epidemiology, in a setting with an integrated PrEP delivery system centred on a public health nurse-led PrEP clinic and referral process.

**Methods:**

This study was conducted in Ottawa, Canada, where all positive HIV test results are reported to the public health units. Risk factor information is also collected by nurses and subsequently entered into a provincial database. We extracted these data for Ottawa from 2017 to 2021 and restricted our analyses to first-time diagnoses.

**Results:**

We identified 154 persons with a new HIV diagnosis. Over this period, the number of new diagnoses among men who have sex with men, the group most targeted for PrEP, decreased by 50%–60%. We did not identify changes in the number of new diagnoses based on race, intravenous drug use or among women.

**Conclusion:**

Increasing PrEP uptake in Ottawa in 2017 to 2021 coincided with a significant decrease in new HIV diagnoses among men who have sex with men. PrEP uptake in Ottawa, particularly by those most at risk, is likely supported by an integrated approach via PrEP-RN, a nurse-led public health program where individuals diagnosed with syphilis or rectal gonorrhea or chlamydia receive an automatic offer of PrEP. While these findings cannot causally link PrEP-RN or PrEP with this reduction in new HIV diagnoses, these changes in HIV epidemiology in Ottawa occurred exclusively among the group targeted for PrEP. These data highlight the efficacy and importance of PrEP.

## Introduction

Beginning with the iPrEx study in 2010 ((1)), evidence has continued to demonstrate the efficacy of emtricitabine plus either tenofovir disoproxil fumarate (DF) (FTC/TDF) or tenofovir alafenamide (AF) (FTC/TAF) as pre-exposure prophylaxis (PrEP) to reduce the risk of human immunodeficiency virus (HIV) infection ((2,3)). These data led the United States Food and Drug Administration to license FTC/TDF for PrEP in 2012, with Health Canada following in 2016. In 2015, the first PrEP clinic launched in Ottawa and targeted men who have sex with men (MSM), who accounted for an estimated 77% of new HIV diagnoses in Ottawa at that time ((4)). Following Health Canada’s approval of PrEP in 2016, two additional community PrEP clinics opened in Ottawa. The first Canadian PrEP guidelines were published in 2017 ((5)).

In 2018, O’Byrne *et al.* ((6,7)) implemented PrEP-RN, a nurse-led PrEP clinic and referral system run by public health nurses. As per provincial public health legislation ((8)), all positive test results for sexually transmitted infections are reported to local health units for follow-up and contact tracing. As part of PrEP-RN, an automatic offer of PrEP was given to anyone diagnosed with infectious syphilis or rectal gonorrhea or chlamydia, or who, based on clinical assessment, was determined to be at risk of HIV infection. Between 2018 and 2021, 1,901 persons fulfilled PrEP-RN eligibility criteria and were offered a referral, of which 49% (n=845/1,736) of eligible persons accepted. Of these 845 persons who accepted PrEP, 95% (n=803) were MSM and 97% (n=820) were male.

These efforts to facilitate PrEP access—from the first clinic in 2015 to our referral system in 2018—led to an increase in the number of persons using PrEP; from 110 in 2016 to over 1,000 in 2021 ((9)). By 2021, this corresponded to a rate of 92/100,000 persons in Ottawa using PrEP ((9)). The use alone of PrEP, however, does not inform whether PrEP uptake is meeting the needs of the province and communities. To do this, it is necessary to evaluate PrEP uptake relative to HIV risk within a population. First-time diagnoses are a proxy for HIV infection and the risk experienced by the community; therefore, we examine PrEP use relative to first-time diagnoses, known as the “PrEP-to-need” ratio ((10,11)). The higher the ratio, the closer PrEP use is meeting the need. The PrEP-to-need ratio also allows comparison across groups and locations to understand PrEP uptake relative to need.

In Ontario, PrEP-to-need ratios have been calculated using commercial pharmacy dispensation data and first-time HIV diagnosis numbers (**Table 1**) ((12)). Corresponding to the reported increased PrEP use *per capita*, in Ottawa, the PrEP-to-need ratio has increased sevenfold, from five in 2017 to 35 in 2021 ((9)). This remains the highest in Ontario and about a third higher than the province overall, after having increased more quickly than elsewhere in Ontario (Table 1) ((9)). Further analyses identified that 97% of persons who use PrEP in Ontario identify as MSM, aligning with PrEP-RN outcomes of most eligible persons being MSM.

**Table 1:** First-time diagnoses, PrEP uptake and PrEP-to-need ratio over time

Year of study	Ontario			Ottawa		
	First-time HIV diagnoses	PrEP users	PrEP-to-need	First-time HIV diagnoses	PrEP users	PrEP-to-need
2017	691	2,998	4.3	51	259	5.1
2018	729	6,543	9.0	43	560	13.0
2019	679	9,797	14.4	34	873	25.7
2020	508	9,584	18.9	37	862	23.3
2021	483	11,005	22.8	27	964	35.7

Abbreviations: HIV, human immunodeficiency virus; PrEP, pre-exposure prophylaxis; PrEP-to-need, PrEP use relative to first-time diagnoses

To understand the impact of our nurse-led PrEP referral and delivery network, and if the increase in the PrEP-to-need ratio in Ottawa corresponded with changes in the number of first-time HIV diagnoses, we undertook a retrospective review of first-time HIV diagnoses in Ottawa between 2017 and 2021. This period was selected because it aligned with the release of the Canadian PrEP guidelines and preceded the implementation of PrEP-RN by 18 months.

## Methods

Positive HIV test results in Ontario are reported to public health units ((8)), including first-time diagnoses, persons undergoing repeat or confirmatory testing, and persons who were previously diagnosed and are undergoing testing for the first time in Ontario. Public health units contact individuals with a positive HIV test to provide counselling, linkage to care and contact tracing. Public health nurses also collect demographic information, including if the individual with the reported HIV positive test result was previously diagnosed with HIV, and age, sex, country of birth and information on risk factors (e.g. sex/drug use practises). The HIV risk factors align with standard HIV data collection and include but are not limited to the following: MSM, report of injection drug use and report of heterosexual contact. Risk factors are treated independently, allowing multiple risks factors to be examined per person. These data are entered into the Integrated Public Health Information System (iPHIS).

### Data collection and analysis

For positive HIV tests reported to Ottawa Public Health from January 1, 2017, to December 31, 2021, we extracted the following from iPHIS: demographic information, including age, ethnicity, sex and country of birth, risk factors, including sex of partners, drug use and prior sexually transmitted infection diagnoses, including a prior HIV diagnosis. We entered these data into a REDCap database and used SAS v.9.4 for analysis. To restrict our analysis to first-time diagnoses, we removed from the dataset any person with a recorded or reported history of an HIV diagnosis prior to their positive test in Ottawa. We assessed associations between demographic characteristics, risk factors and year of diagnosis using chi-square tests. The HIV risk factors were tested independently, as follows: male to male sexual contact versus no reported male to male sexual contact, injection drug use versus no reported injection drug use, and heterosexual contact versus no reported heterosexual contact. The HIV risk factors were not treated as mutually exclusive. Because all HIV diagnostic testing in Ontario is carried out by the Public Health Ontario Laboratories, we obtained the total number of HIV tests performed by demographic and location in the province ((13)). We calculated test positivity by dividing the number of first-time diagnoses by the number of tests in Ottawa (overall, by birth sex, and for MSM) by year (excluding prenatal tests). We analyzed trends in test positivity over time using a Cochran-Armitage test.

## Results

In Ottawa, from January 1, 2017, to December 31, 2021, we identified 154 people diagnosed for the first time with HIV (**Table 2**). Of these, 41 new diagnoses were documented in 2017, 34 in 2018 followed by a progressive decline that levelled off to 26–27 new diagnoses per year in 2019–2021. This is a 37% decline in overall new diagnoses in 2021, compared to 2017. A chi-square test was used to determine whether diagnosis counts changed significantly over the time by demographic characteristic or HIV risk factor. The apparent decline in first-time diagnoses was significant only among men (*p*<0.01) and MSM (*p*<0.05) (Table 2). Moreover, 19 MSM were newly diagnosed with HIV in 2017, 16 in 2018, and only 5–8 diagnoses occurred in this group each year in 2019–2021, representing a 57% drop between 2017 and 2021. We did not see any significant change in the number of new HIV diagnoses over the study period among those who reported heterosexual contact compared to those that did not (p=0.68), those who reported using intravenous drugs compared to those that did not (p=0.19) or females compared to males (p=0.09). We also did not identify any change in the number of first-time HIV diagnoses based on race/ethnicity (Black versus White) or age (younger than 35 years or 35 years and older) (Table 2).

**Table 2:** Human immunodeficiency virus diagnoses over time

Demographic/risk factors	N (%)						*p*-value
	2017	2018	2019	2020	2021	Total	
Total	41	34	26	27	26	154	N/A
**Birth sex**							
Male	34 (83%)	26 (76%)	14 (54%)	16 (59%)	17 (65%)	107 (69%)	0.0899
Female	7 (17%)	8 (24%)	12 (46%)	11 (41%)	8 (31%)	46 (30%)	N/A
**HIV risk factors**							
MSM	19 (46%)	16 (47%)	6 (23%)	5 (19%)	8 (31%)	54 (35%)	0.049
Not MSM	22 (54%)	18 (53%)	20 (77%)	22 (81%)	18 (69%)	100 (65%)	N/A
IDU	8 (20%)	8 (24%)	1 (4%)	8 (30%)	6 (23%)	31 (20%)	0.1848
Not IDU	33 (80%)	26 (76%)	25 (96%)	19 (70%)	20 (77%)	123 (80%)	N/A
Heterosexual^a^	21 (51%)	16 (47%)	17 (65%)	19 (70%)	13 (50%)	86 (56%)	0.6822
Not heterosexual	17 (41%)	11 (32%)	9 (35%)	8 (30%)	12 (46%)	57 (37%	N/A
**Race/ethnicity**							
Black	18 (44%)	6 (18%)	16 (62%)	10 (37%)	9 (35%)	59 (38%)	0.059
White	17 (41%)	22 (65%)	7 (27%)	13 (48%)	14 (54%)	73 (47%)	N/A
Other	3 (7%)	3 (9%)	1 (4%)	4 (15%)	2 (8%)	13 (8%)	N/A
**Age category (years)**							
Younger than 35	16 (39%)	11 (32%)	8 (31%)	12 (44%)	6 (23%)	53 (34%)	0.511
35 and older	25 (61%)	23 (68%)	18 (69%)	15 (56%)	20 (77%)	101 (66%)	N/A

Abbreviations: HIV, human immunodeficiency virus; IDU, people who reported injection drug use; MSM, men who have sex with men; N/A, not applicable

^a^ Heterosexual means people who reported heterosexual contact

As diagnoses may have been affected by decreased testing during the coronavirus disease 2019 (COVID-19) pandemic, we analyzed trends in test positivity in Ottawa over the same period. If there had been no change in the rate of HIV transmission and the decrease in new diagnoses was due to decreased testing, the test positivity rate should have remained unchanged. Here we examined test positivity overall, by birth sex and for MSM (**Table 3**). While there was a small decrease in the test positivity overall, from 0.07% in 2017 to 0.04% in 2021, there was a significant decrease only in men (*p*<0.05) and MSM (*p*<0.01), suggesting a true reduction in HIV transmission. We did not identify significant changes in the test positivity rate for women (*p*=0.27) (Table 3).

**Table 3:** Human immunodeficiency virus test positivity over time

Demographic/risk factors	Year					Trend test *p*-value
	2017	2018	2018	2020	2021	
Overall	0.07	0.05	0.05	0.06	0.04	0.07
**Birth sex**						
Male	0.11	0.08	0.05	0.07	0.04	0.002
Female	0.02	0.03	0.04	0.05	0.03	0.07
**HIV risk**						
MSM	0.37	0.4	0.16	0.18	0.18	0.02

Abbreviations: HIV, human immunodeficiency virus; MSM, men who have sex with men

## Discussion

We report here a significant decrease ((1)) in the HIV test positivity among men and MSM in Ottawa and ((2)) in the number of first-time HIV diagnoses in MSM from 2017 to 2021. From 2012–2016, the rolling average of new HIV diagnoses each year among MSM in Ottawa was 31.3 (range: 21–40) ((14)). In 2017, there were 19 new HIV infections in Ottawa in this group and in 2019–2021, this number dropped to 5–8 new diagnoses per year. Coincident with this decrease was the progressive increase in the PrEP-to-need ratio ((15,16)).

While we cannot prove causality, the link between increasing PrEP uptake (as evidenced by absolute increase and an increasing PrEP-to-need ratio; Table 1) and the decrease in HIV incidence in Ottawa is inferred by the fact that MSM were targeted for PrEP and it was in this group only that the number of first-time HIV diagnoses decreased. That we did not see a decrease among women or persons who use intravenous drugs, groups where PrEP uptake has been low in Ottawa and among whom PrEP was not as well targeted as part of PrEP-RN, supports the link between an increase in PrEP use and a decrease in the number of first-time HIV diagnoses. These data align with research from Australia ((17)), Scotland ((18)), Uganda and Kenya ((19)) and the United States ((20)), which have documented decreased HIV incidence after implementing high-coverage access to PrEP for MSM.

We do not believe the decreasing trend in new HIV diagnoses in Ottawa can be attributed to COVID-19 and reduced testing. First, the decrease in the absolute number of new diagnoses and in test positivity among MSM started prior to the pandemic (coincident with the launch of PrEP-RN) and was sustained over the next two years (with preliminary analyses of 2022 data showing the decrease was sustained for a third year). Second, the decrease in new diagnoses was essentially restricted to MSM, the group targeted for PrEP and where uptake was greatest. That the number of new HIV diagnoses did not change over this period for persons who use intravenous drugs or women provides a comparator group. Had decreased access to testing caused the decrease in diagnoses, one would predict a broader decrease in HIV incidence including in other demographic groups. Third, had the decrease in new diagnoses been due to decreased testing, the change in test positivity would not have shown a significant decline over the study period. Test positivity was unchanged for women, while there was a significant decrease among men, among whom MSM experienced the greatest decline.

It is equally unlikely that the outcomes we observed are related to HIV treatment ((21)), through which HIV-positive people can achieve undetectable viral loads and untransmittable infections (i.e. undetectable equals untransmittable, or U=U). In Ontario, Ottawa has the second-highest rate of persons living with HIV and the second-lowest rate of engagement in HIV care ((22)). Alternate explanations for the decreased number of new HIV infections we observed include 1) that the prevalence of HIV infection was too low for transmission to have occurred and 2) that there was no opportunity for transmission due to high levels of viral suppression among persons living with HIV. However, neither appears true in Ottawa. Without a change in testing and given that the reduction in HIV was confined to MSM, increasing PrEP uptake is the major factor that changed during our study.

Our data raise a few points for discussion. The first is the PrEP-to-need ratio and its relationship to the number of first-time HIV diagnoses. As noted, in Ottawa, the PrEP-to-need ratio increased from 5 in 2017 to 35 in 2021 ((9,16)) and, notably, the drop in first-time HIV diagnoses among MSM occurred in 2019 and was sustained thereafter. This raises the question of whether there is a potential PrEP-to-need threshold that coincides with substantial decreases in new HIV infections. The concept would be akin to herd immunity and represents a point where enough people use PrEP to prevent ongoing HIV transmission. If such a threshold exists, it is very unlikely to be a single target and most likely will vary depending on the transmission network and ecological context, plus whether PrEP was deployed generally or in the targeted fashion offered by PrEP-RN. Further research is absolutely required.

Second, our data suggest a potentially efficient way of addressing the initial steps of the PrEP cascade; specifically, identifying individuals at risk of HIV infection, making an offer of PrEP and linking those who accept care. While we very strongly support increased and broad community awareness as well as increased PrEP capacity in primary care, public health units are uniquely situated to reach those individuals at greatest risk of HIV infection ((23)). Table 1 shows the increasing PrEP-to-need ratio in Ottawa that occurred after the implementation of targeted and systematic PrEP recommendations by public health nurses, demonstrating that the PrEP-to-need ratio increased in Ottawa faster than across Ontario, in part due to PrEP-RN implementation. Virtually all extant PrEP guidelines ((4,24,25)) recommend an offer of PrEP to anyone diagnosed with infectious syphilis or rectal gonorrhea or chlamydia, and to the sexual partners of someone with transmissible HIV. As studies have identified HIV diagnosis rates of 7%–8% within one year of these indicators ((24)), it follows that HIV incidence would decrease after a public health unit implemented a program that offered PrEP to those meeting these criteria. This is made possible by the fact that all positive sexually transmitted infection results are reported to public health units in Ontario. This creates a feasible, high-yield strategy with a potentially low number-needed-to-treat. Further, that the decrease in HIV diagnoses in this study was observed in MSM reinforces the validity of the indicator criteria by showing how targeted recommendations for, and provisions of, PrEP can coincide with decreases in the number of first-time HIV diagnoses among MSM at the population level.

Third, despite the apparent benefits of using the current indicator criteria for initiating PrEP among MSM, our findings support criticisms highlighting a lack of emphasis on HIV risk factors for people who use injection drugs and for heterosexual exposures, which disproportionately affects Black and Indigenous Peoples, potentially exacerbating existing health disparities for these groups ((26–30)). Furthermore, evidence has shown that risk factors do not correlate directly across groups, with Black MSM experiencing a greater risk of HIV transmission, while reporting fewer risk factors than White counterparts ((30)). Our findings, which showed a significant decrease in new HIV diagnoses among MSM but not for other groups, support this criticism. While PrEP-RN was not exclusively restricted to MSM, it did in effect target these men as current guidelines for PrEP ((4,25–27)) best serve these men, thus enabling this population to experience a greater uptake of PrEP. Concerted efforts are now required to determine PrEP indicators for other populations, thus enabling other groups to benefit from the potential population-level effects we observed among MSM in Ottawa.

## Limitations

First, our data were based on reported positive HIV test results, which relies on people accessing testing. Among those with new diagnoses, time may have passed since transmission occurred, so temporality is difficult to establish. However, this is not a new limitation of HIV epidemiology and the sustained decrease in diagnoses suggests a decline in HIV incidence. Second, COVID-19 became widespread in 2020 and so the decreased number of new HIV diagnoses could have resulted from reduced sexual activity or testing. Since we observed these decreases beginning in 2019, we think this is unlikely. It is equally unlikely that COVID-19 would have affected changes in the PrEP-to-need ratio exclusively in Ottawa, compared to across Ontario (see Table 1). Third, our data regarding risk factors was based on self-report to public health nurses, although this has been the historical approach to data collection for HIV epidemiology, and so would not have manifested as a change in our data compared to those preceding them. Nonetheless, because the variables in our analysis were limited to the data collected by public health nurses, there were potential confounders or effect modifiers not examined in this analysis. Lastly, our data arose from one city in Canada without comparison. It is possible that the decreases in new diagnoses are related to influences that we have yet to identified. While possible, to the best of our knowledge, there were no other major changes related to, or interventions targeted at, HIV, MSM or other at-risk populations in Ottawa during the look-back period. We also know there were no changes in the uptake of HIV treatment or in levels of viral suppression in our region during this time. Our data also show a comparison in PrEP-to-need ratios across Ontario, identifying a faster increase in PrEP uptake in Ottawa in conjunction with PrEP-RN implementation.

## Conclusion

We report here on a significant decrease in the number of first-time HIV diagnoses and in HIV test positivity in Ottawa from 2017 to 2021 among MSM, coincident with increased PrEP uptake within this group (as evidence by increasing PrEP-to-need ratios in Ottawa). While our results cannot show causality, decreased diagnoses occurring only in the groups targeted for PrEP (men and MSM) suggests a relationship. As the reduction in HIV diagnoses was first noted in 2019, and because the HIV test positivity rate dropped for MSM but no other group, we do not believe the effect was the result of COVID-19 or changes in access to healthcare. We also note that as the PrEP-to-need ratio increased from 2017 to 2021 (primarily among MSM), the greatest decrease in new HIV diagnoses occurred in 2019. While our analyses highlighted the utility of using the PrEP-to-need ratio as part of understanding overall PrEP uptake and HIV diagnosis numbers, a question for ongoing research is the possibility that there is a PrEP-to-need threshold that must be reached to prevent HIV transmission. Finally, the focus on individuals with a diagnosis of syphilis or rectal gonorrhea or chlamydia as most in need of PrEP potentially restricted the benefits of this intervention to MSM. Future work needs to elucidate guidelines for people who use injection drugs and those with heterosexual risk factors that account for differential population-level risk, with the specific intent to improve health equity for Black and Indigenous Peoples. While our results emerge from small numbers, they nevertheless constitute important data on the key role public health units can play in the initial steps of the PrEP cascade, the strength of existing criteria to identify those who would benefit from PrEP, and the need to better understand HIV risk in other populations. Indeed, these results provide a proof-of-concept that systematically offering PrEP may lead to a decrease in HIV incidence in MSM, driven by the targeting of PrEP to high-risk persons. This is part of public health follow-up for infectious syphilis, rectal gonorrhea and chlamydia, as it was done for PrEP-RN. With such ongoing efforts, PrEP will no doubt reduce ongoing HIV transmission, improving both individual and population health.

## References

[1] Grant RM, Lama JR, Anderson PL, McMahan V, Liu AY, Vargas L, Goicochea P, Casapía M, Guanira-Carranza JV, Ramirez-Cardich ME, Montoya-Herrera O, Fernández T, Veloso VG, Buchbinder SP, Chariyalertsak S, Schechter M, Bekker LG, Mayer KH, Kallás EG, Amico KR, Mulligan K, Bushman LR, Hance RJ, Ganoza C, Defechereux P, Postle B, Wang F, McConnell JJ, Zheng JH, Lee J, Rooney JF, Jaffe HS, Martinez AI, Burns DN, Glidden DV; iPrEx Study Team. Preexposure chemoprophylaxis for HIV prevention in men who have sex with men. N Engl J Med 2010;363(27):2587–99. 10.1056/NEJMoa1011205 21091279 PMC3079639

[2] McCormack S, Dunn DT, Desai M, Dolling DI, Gafos M, Gilson R, Sullivan AK, Clarke A, Reeves I, Schembri G, Mackie N, Bowman C, Lacey CJ, Apea V, Brady M, Fox J, Taylor S, Antonucci S, Khoo SH, Rooney J, Nardone A, Fisher M, McOwan A, Phillips AN, Johnson AM, Gazzard B, Gill ON. Pre-exposure prophylaxis to prevent the acquisition of HIV-1 infection (PROUD): effectiveness results from the pilot phase of a pragmatic open-label randomised trial. Lancet 2016;387(10013):53–60. 10.1016/S0140-6736(15)00056-2 26364263 PMC4700047

[3] Mayer KH, Molina JM, Thompson MA, Anderson PL, Mounzer KC, De Wet JJ, DeJesus E, Jessen H, Grant RM, Ruane PJ, Wong P, Ebrahimi R, Zhong L, Mathias A, Callebaut C, Collins SE, Das M, McCallister S, Brainard DM, Brinson C, Clarke A, Coll P, Post FA, Hare CB. Emtricitabine and tenofovir alafenamide vs emtricitabine and tenofovir disoproxil fumarate for HIV pre-exposure prophylaxis (DISCOVER): primary results from a randomised, double-blind, multicentre, active-controlled, phase 3, non-inferiority trial. Lancet 2020;396(10246):239–54. 10.1016/S0140-6736(20)31065-5 32711800 PMC9665936

[4] Spatz Friedman D, O’Byrne P, Roy M. Comparing those diagnosed early versus late in their HIV infection: implications for public health. Int J STD AIDS 2017;28(7):693–701. 10.1177/0956462416664674 27538724

[5] Tan DH, Hull MW, Yoong D, Tremblay C, O’Byrne P, Thomas R, Kille J, Baril JG, Cox J, Giguere P, Harris M, Hughes C, MacPherson P, O’Donnell S, Reimer J, Singh A, Barrett L, Bogoch I, Jollimore J, Lambert G, Lebouche B, Metz G, Rogers T, Shafran S; Biomedical HIV Prevention Working Group of the CIHR Canadian HIV Trials Network. Canadian guideline on HIV pre-exposure prophylaxis and nonoccupational postexposure prophylaxis. CMAJ 2017;189(47):E1448–58. 10.1503/cmaj.170494 29180384 PMC5703677

[6] OʼByrne P, MacPherson P, Orser L, Jacob JD, Holmes D. PrEP-RN: clinical considerations and protocols for nurse-led PrEP. J Assoc Nurses AIDS Care 2019;30(3):301–11. 10.1097/JNC.0000000000000075 31008817 PMC6551248

[7] O’Byrne P, Vandyk A, Orser L, Haines M. Nurse-led PrEP-RN clinic: a prospective cohort study exploring task-Shifting HIV prevention to public health nurses. BMJ Open 2021;11(1):e040817. 10.1136/bmjopen-2020-040817 33414144 PMC7797243

[8] Government of Ontario. Health Protection and Promotion Act, R.S.O. 1990, c. H.7. https://www.ontario.ca/laws/statute/90h07

[9] Ontario HIV Treatment Network. HIV Pre-Exposure Prophylaxis (PrEP) in Ontario, 2020. Toronto, ON: OHTH, 2020. [Accessed 2022 June 10]. https://www.ohesi.ca/wp-content/uploads/2022/03/OHTN-PrEP-report-2020_vf.pdf

[10] Siegler AJ, Mouhanna F, Giler RM, Weiss K, Pembleton E, Guest J, Jones J, Castel A, Yeung H, Kramer M, McCallister S, Sullivan PS. The prevalence of pre-exposure prophylaxis use and the pre-exposure prophylaxis-to-need ratio in the fourth quarter of 2017, United States. Ann Epidemiol 2018;28(12):841–9. 10.1016/j.annepidem.2018.06.005 29983236 PMC6286209

[11] Sullivan PS, Giler RM, Mouhanna F, Pembleton ES, Guest JL, Jones J, Castel AD, Yeung H, Kramer M, McCallister S, Siegler AJ. Trends in the use of oral emtricitabine/tenofovir disoproxil fumarate for pre-exposure prophylaxis against HIV infection, United States, 2012-2017. Ann Epidemiol 2018;28(12):833–40. 10.1016/j.annepidem.2018.06.009 30037634 PMC6286244

[12] Tan DH, Dashwood TM, Wilton J, Kroch A, Gomes T, Martins D. Trends in HIV pre-exposure prophylaxis uptake in Ontario, Canada, and impact of policy changes: a population-based analysis of projected pharmacy data (2015-2018). Can J Public Health 2021;112(1):89–96. 10.17269/s41997-020-00332-3 32529552 PMC7851246

[13] Ontario HIV Epidemiology and Surveillance Initiative. HIV Testing in Ontario, 2019. Toronto, ON: OHESI; 2021. https://www.ohesi.ca/wp-content/uploads/2021/10/2019-HIV-Testing-in-Ontario.pdf

[14] Ontario HIV Epidemiology and Surveillance Initiative. HIV Diagnoses in Ontario, 2020. Tables Supplement. Toronto, ON: OHESI; 2022. https://www.ohesi.ca/wp-content/uploads/2022/08/HIV-diagnoses-in-Ontario-2020-TABLES-Supplement-1.pdf

[15] Ontario HIV Epidemiology and Surveillance Initiative. HIV diagnoses in Ontario, 2020. 2023. https://www.ohesi.ca/wp-content/uploads/2022/08/HIV-diagnoses-in-Ontario-2020-REPORT-FINAL-1.pdf

[16] Ontario HIV Treatment Network. HIV pre-exposure prophylaxis Ontario (PrEP), 2021. 2023. https://www.ohtn.on.ca/wp-content/uploads/2023/04/OHTN-PrEP-report-2021-2023APR06.pdf

[17] Grulich AE, Guy R, Amin J, Jin F, Selvey C, Holden J, Schmidt HA, Zablotska I, Price K, Whittaker B, Chant K, Cooper C, McGill S, Telfer B, Yeung B, Levitt G, Ogilvie EE, Dharan NJ, Hammoud MA, Vaccher S, Watchirs-Smith L, McNulty A, Smith DJ, Allen DM, Baker D, Bloch M, Bopage RI, Brown K, Carr A, Carmody CJ, Collins KL, Finlayson R, Foster R, Jackson EY, Lewis DA, Lusk J, O’Connor CC, Ryder N, Vlahakis E, Read P, Cooper DA; Expanded PrEP Implementation in Communities New South Wales (EPIC-NSW) research group. Population-level effectiveness of rapid, targeted, high-coverage roll-out of HIV pre-exposure prophylaxis in men who have sex with men: the EPIC-NSW prospective cohort study. Lancet HIV 2018;5(11):e629–37. 10.1016/S2352-3018(18)30215-7 30343026

[18] Estcourt C, Yeung A, Nandwani R, Goldberg D, Cullen B, Steedman N, Wallace L, Hutchinson S. Population-level effectiveness of a national HIV preexposure prophylaxis programme in MSM. AIDS 2021;35(4):665–73. 10.1097/QAD.0000000000002790 33290298 PMC7924973

[19] Koss CA, Havlir DV, Ayieko J, Kwarisiima D, Kabami J, Chamie G, Atukunda M, Mwinike Y, Mwangwa F, Owaraganise A, Peng J, Olilo W, Snyman K, Awuonda B, Clark TD, Black D, Nugent J, Brown LB, Marquez C, Okochi H, Zhang K, Camlin CS, Jain V, Gandhi M, Cohen CR, Bukusi EA, Charlebois ED, Petersen ML, Kamya MR, Balzer LB. HIV incidence after pre-exposure prophylaxis initiation among women and men at elevated HIV risk: A population-based study in rural Kenya and Uganda. PLoS Med 2021;18(2):e1003492. 10.1371/journal.pmed.1003492 33561143 PMC7872279

[20] Smith DK, Sullivan PS, Cadwell B, Waller LA, Siddiqi A, Mera-Giler R, Hu X, Hoover KW, Harris NS, McCallister S. Evidence of an Association of Increases in Pre-exposure Prophylaxis Coverage With Decreases in Human Immunodeficiency Virus Diagnosis Rates in the United States, 2012-2016. Clin Infect Dis 2020;71(12):3144–51. 10.1093/cid/ciz1229 32097453 PMC7819523

[21] United States Department of Health & Human Services. HIV Treatment as Prevention. HIV.gov; 2023. [Accessed 2023 Jan 30]. https://www.hiv.gov/tasp

[22] Ontario HIV Epidemiology and Surveillance Initiative. HIV care cascade in Ontario: Linkage to care, in care, on antiretroviral treatment, and virally suppressed, 2020. Toronto, ON: OHESI; Oct 4, 2022. [Accessed 2023 Jan 20]. https://www.ohesi.ca/wp-content/uploads/2022/10/HIV-Care-Cascade-2020-final-1.pdf

[23] Orser L, O’Byrne P. The role of public health units in the delivery of HIV pre-exposure prophylaxis (PrEP). Can J Public Health 2019;110(1):72–5. 10.17269/s41997-018-0141-7 30341481 PMC6964540

[24] Centers for Disease Control and Prevention. Preexposure prophylaxis for the prevention of HIV infection in the United States – 2021 Update: A Clinical Practice Guideline. Atlanta, GA; CDC; 2021. [Accessed 2022 Jan 30]. https://www.cdc.gov/hiv/pdf/risk/prep/cdc-hiv-prep-guidelines-2021.pdf

[25] World Health Organization. Consolidated guidelines on the use of antiretroviral drugs for treating and preventing HIV infection: recommendations for a public heath approach, 2nd ed. Geneva (CH): WHO; 2016. [Accessed 2022 Jan 30]. https://www.who.int/publications/i/item/9789241549684 27466667

[26] Nelson LE, James L, Coleman T, Etowa J, Husbands W, Lofters A, Mitchell MO, Nguemo JD, Nnorom O, Oraka C, Rana J, Siddiqi A, Wilson CL. A recipe for increasing racial and gender disparities in HIV infection: A critical analysis of the Canadian guideline on pre-exposure prophylaxis and non-occupational post-exposure prophylaxis’ responsiveness to the HIV epidemics among women and Black communities. Can J Hum Sex 2019;28(1):1–4. 10.3138/cjhs.2018-004325382990

[27] Adams JW, Khan MR, Bessey SE, Friedman SR, McMahon JM, Lurie MN, Galea S, Marshall BD. Preexposure prophylaxis strategies for African-American women affected by mass incarceration. AIDS 2021;35(3):453–62. 10.1097/QAD.0000000000002749 33170818 PMC7855567

[28] Garnett M, Hirsch-Moverman Y, Franks J, Hayes-Larson E, El-Sadr WM, Mannheimer S. Limited awareness of pre-exposure prophylaxis among black men who have sex with men and transgender women in New York city. AIDS Care 2018;30(1):9–17. 10.1080/09540121.2017.1363364 28791876 PMC5699928

[29] Mayer KH, Agwu A, Malebranche D. Barriers to the wider use of pre-exposure prophylaxis in the United States: A narrative review. Adv Ther 2020;37(5):1778–811. 10.1007/s12325-020-01295-0 32232664 PMC7467490

[30] Millett GA, Peterson JL, Wolitski RJ, Stall R. Greater risk for HIV infection of black men who have sex with men: a critical literature review. Am J Public Health 2006;96(6):1007–19. 10.2105/AJPH.2005.066720 16670223 PMC1470628

